# Imaging in assessing hepatic and peritoneal metastases of gastric cancer: a systematic review

**DOI:** 10.1186/1471-230X-11-19

**Published:** 2011-03-09

**Authors:** Zhen Wang, Jun-Qiang Chen

**Affiliations:** 1 Department of Gastrointestinal Surgery, The First Affiliated Hospital of Guangxi Medical University, 6 Shuang Yong Road, Nanning 530021, Guangxi, PR China

## Abstract

**Background:**

Hepatic and peritoneal metastases of gastric cancer are operation contraindications. Systematic review to provide an overview of imaging in predicting the status of liver and peritoneum pre-therapeuticly is essential.

**Methods:**

A systematic review of relevant literatures was performed in Pubmed/Medline, Embase, The Cochrane Library and the China Biological Medicine Databases. QUADAS was used for assessing the methodological quality of included studies and the bivariate model was used for this meta-analysis.

**Results:**

Totally 33 studies were included (8 US studies, 5 EUS studies, 22 CT studies, 2 MRI studies and 5 18F-FDG PET studies) and the methodological quality of included studies was moderate. The result of meta-analysis showed that CT is the most sensitive imaging method [0.74 (95% CI: 0.59-0.85)] with a high rate of specificity [0.99 (95% CI: 0.97-1.00)] in detecting hepatic metastasis, and EUS is the most sensitive imaging modality [0.34 (95% CI: 0.10-0.69) ] with a specificity of 0.96 (95% CI: 0.87-0.99) in detecting peritoneal metastasis. Only two eligible MRI studies were identified and the data were not combined. The two studies found that MRI had both high sensitivity and specificity in detecting liver metastasis.

**Conclusion:**

US, EUS, CT and ^18^F-FDG PET did not obtain consistently high sensitivity and specificity in assessing liver and peritoneal metastases of gastric cancer. The value of laparoscopy, PET/CT, DW-MRI, and new PET tracers such as ^18^F-FLT needs to be studied in future.

## Background

Although the decreasing incidence and mortality, gastric cancer remains the fourth common cancer and the second leading cause of cancer-related deaths with poor prognosis worldwide [1,2]. As we known, treatment option, decision-making and prognosis of gastric cancer are strongly dependent on the extent of tumor (tumor extension, nodal involvement and distant metastases), accurately pretherapeutic staging is essential [3].

It was reported that the rate of liver metastasis in gastric cancer can achieve 5-9% [4,5], and the number of liver metastasis is a significant prognostic factor of gastric cancer [4,6]. Generally speaking, gastric cancer has extrahepatic metastasis if hepatic metastasis, such as lymph node involvement and peritoneal seeding. Surgical resection is rarely required under these circumstances [5]. Peritoneal metastasis, mainly induced by the dissemination of free tumor cells from the primary gastric cancer, is one of the most common types of spread and the causes of death [7]. Peritoneal metastasis of gastric cancer was considered to be operation contraindication and the most difficult type for treatment [8].

Studies suggested that imaging methods of evaluating the pre-operative status of hepatic and peritoneal metastases have two effects [9,10]: 1) avoiding unnecessary laparotomy; 2) assessing the effectiveness of neoadjuvant protocols in the absence of histopathological confirmation. Although systematic review and meta-analysis of imaging in assessing local staging and lymph node status of gastric cancer were performed [11,12], there is no consensus on the most sensitive imaging method for detecting hepatic and peritoneal metastases of gastric cancer now. Theoretically pre-operative staging of gastric cancer should mainly focus on assessing distant metastases but not local staging or lymph node status, since if one patient has distant metastases, an exploratory laparotomy always can be avoided [13].

The objective of this systematic review is to provide a comprehensive and up-to-date overview of sensitivity and specificity of imaging [ultrasonography(US), Endoscopic ultrasound(EUS), computed tomography (CT), magnetic resonance imaging(MRI), and ^18^F-fluorodeoxyglucose positron emission tomography(^18^F-FDG PET)] in detecting hepatic and peritoneal metastases of gastric cancer.

## Methods

### Search strategy

A computer-aided search of the Pubmed/Medline, Embase, The Cochrane Library (issue 1, 2011), and the China Biological Medicine Database (CBM) was conducted to identify relevant publications on the diagnostic performance of imaging (US, EUS, CT, MRI, and ^18^F-FDG PET) in detecting hepatic and peritoneal metastases of gastric cancer. The upper limit of search date was not limited, and the lower limit was February, 2011. The following search phrases were used: stomach neoplasms, stomach cancer, stomach carcinoma, stomach tumor, gastric cancer, gastric carcinoma, gastric neoplasms, gastric tumor, liver metastasis/metastases, hepatic metastasis/metastases, peritoneal metastasis/metastases, peritoneal seeding, peritoneal involvement, peritoneal carcinomatosis, sensitivity, specificity, accuracy. Both free text and MeSH search for keywords were employed. The language was not limited. To search more potentially relevant trials, reference lists from included studies of electronic searching were screened.

### Inclusion and exclusion criteria

Inclusion criteria for this meta-analysis

1. Studies assessed the diagnostic value of imaging (US, EUS, CT, MRI, or ^18^F-FDG PET) in detecting hepatic or peritoneal metastasis of gastric cancer.

2. The standard of reference had to be a surgery or histopathological examination.

3. True-positive, false-positive, true-negative, and false-negative results of imaging methods could be calculated for per-patient.

4. PET had to be performed with intravenous administration of ^18^F-FDG.

Exclusion criteria for this meta-analysis

1. Studies included patients with non-adenocarcinoma (eg, lymphoma).

2. Studies only assessed gastric cancer confined to a specific part of the stomach (eg, cardia or gastroesophageal junction), which could not represent overall place where tumour may occur.

3. Studies included patients who received radiotherapy or chemotherapy pre-operatively, which may cause downstaging. (Because neoadjuvant protocols can lead to tumor downstaging and affect the diagnostic accuracy of imaging)

4. Vitro studies and studies performed in animals.

5. Studies with a sample size less than 10.

6. Studies were not original research (eg, systematic review)

Study selection was performed by two authors (Z. Wang and J.Q.Chen) independently according to the inclusion and exclusion criteria. When we found eligible studies with published data more than once, we only included the article with the most patients. Disagreements were resolved by consensus.

### Data extraction and quality assessment

Two authors (Z. Wang and J.Q.Chen) extracted data using pre-defined tables, which included items as follows: author and publication time, country of source, sample size, interpreters, standard reference, image modality (US, EUS, CT, MRI, or ^18^F-FDG PET), imaging technique (transducer frequency for US and EUS; use of intravenous contrast, section thickness and gap for CT; use of intravenous contrast, section thickness, gap, field strength and coil type for MRI; time of fasting before scanning, FDG dose, time interval between FDG administration and scanning, attenuation correction, and reconstruction method for ^18^F-FDG PET) and test result (true positive, false positive, true negative and false negative on per patient basis).

Fourteen items of QUADAS were used to assess the methodological quality of eligible studies [14]. Descriptions of each item: Yes (score 2); Unclear (score 1); No (score 0). Total quality score was the summary score of each item. We consider that studies with a total score more than 17 were regarded as high methodological quality, and less than 17 as low methodological quality.

### Data analysis

Pooled estimates of sensitivity, specificity and diagnostic odds ratio (DOR) of imaging (with corresponding 95% confidence intervals [CIs]) were analyzed based on the bivariate model [15]. The bivariate model uses a random effect approach for both sensitivity and specificity, which allows for heterogeneity beyond chance as a result of clinical and methodological differences between studies, and the bivariate model is considered as a more valid statistical model for diagnostic meta-analysis [16,17]. To graphically present the results, we plotted the hierarchical summary receiver operating characteristic (HSROC) curves [16]. As a potential cause of heterogeneity in sensitivity and specificity among the included studies, threshold/cut off effect was tested with the Spearman correlation coefficient between the logit of sensitivity and logit of 1-specificity; heterogeneity induced by factors other than threshold/cut off effect was assessed by using the Cochran Q statistic (χ^2 ^test). Statistical significance of heterogeneity test was assumed when a *P *value was less than 0.10. As a concern for meta-analysis of diagnostic trials, publication bias was tested using the funnel plot and Deeks test [18], which was conducted by a regression of diagnostic log odds ratio against 1/sqrt(effective sample size), weighting by effective sample size, with *P *< 0.10 for the slope coefficient indicating significant asymmetry. Meta-Disc (version 1.4), Stata (version 11.0), especially the midas and metandi commands were used for statistical analysis [19,20] (Appendix).

## Results

### Study selection and description

**Figure 1:**
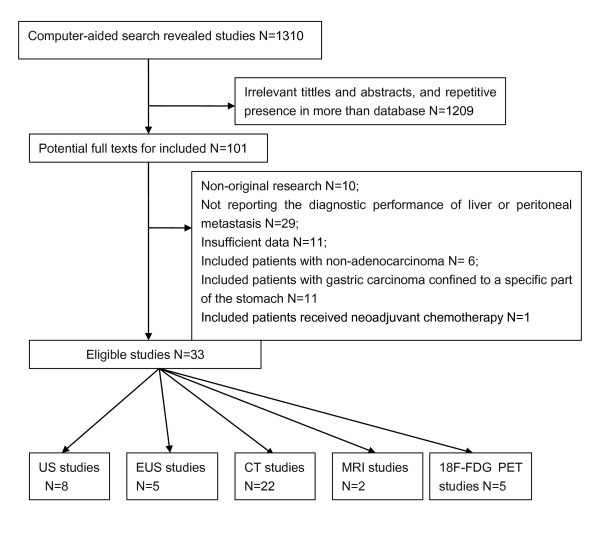
QUORUM flow chart for including studies.

According to the pre-defined search strategy, total 1310 literatures were revealed: 449 from Pubmed/Medline, 853 from Embase, 0 from the Cochrane Library and 8 from CBM. By screening the tittles and abstracts we found that lots of articles were irrelevant and some were identified in more than one database, thus 101 studies remained for potential inclusion and were obtained in full-text version. After reviewing the full text, 68 studies were excluded. The mainly reasons for excluded studies were as follows: non-original research(eg. review articles), not reporting the diagnostic performance of liver or peritoneal metastasis, insufficient data to construct a 2 × 2 contingency table, inclusion of patients with non-adenocarcinoma, gastric carcinoma confined to a specific part of the stomach or included patients received neoadjuvant chemotherapy. At last 33 studies [21-53] were included (8 US studies, 5 EUS studies, 22 CT studies, 2 MRI studies and 5 ^18^F-FDG PET studies). The process of study selection was listed in Figure 1.

The 8 US studies were published between 1983 and 2004, and the sample size varied from 21 to 125 (Table 1). The 5 EUS studies were published between 1990 and 2005, and the sample size ranged from 48 to 402 (Table 2). The 22 CT studies were published between 1994 and 2010, and the sample size varied from 36 to 640 (Table 3). The 2 MRI studies were published between 2006 and 2007, and the sample size varied from 25 to 35 (Table 4). The 5 ^18^F-FDG PET studies were published between 1998 and 2006, and the sample size varied from 23 to 124. Among the 5 ^18^F-FDG PET studies, one study [35] used two kinds of scanners (PT931/04 scanner and SET2400W scanner), and we analyzed the data separatedly according to the kind of scanner (Table 5).

**Table 1:** Characteristics of the eight included US studies

Study (year, reference)	County	No. of patients	Transducer frequency (MHz)	Interpreter(s)	Reference standard
Kim 1997 [21]	South Korea	95	3.5 or 5	Two radiologists independently	Surgical and pathological findings
Stell 1996 [22]	United Kingdom	103	3.5	Experienced personnel	Histological examination
Asencio 1997 [23]	Germany	71	NCD	NCD	Surgical and histologic evaluation
Possik 1986 [24]	United States	82	NCD	NCD	Surgical and histologic evaluation
Derchi 1983 [25]	United States	21	3.5	Two authors of the study	Surgical and pathological examization.
Liao 2004 [26]	China	125	3.5 or 6.0	NCD	Operative and pathological examination
Ozmen 2003 [27]	United States	48	NCD	NCD	Histological examination
Kayaalp 2002 [28]	United Kingdom	118	NCD	A consultant radiologist	Histopathological examinations

NCD: not clearly described

**Table 2:** Characteristics of the five included EUC studies

Study (year, reference)	County	No. of patients	Transducer frequency (MHz)	Interpreter(s)	Reference standard
Ozmen 2003 [27]	United States	48	NCD	NCD	Histological examination
Chu 2004 [30]	United States	402	12	An author of the study	Histopathologic examination
Tio 1990 [31]	United States	84	7.5 or 12	NCD	Surgical and pathological examization.
Chen 2002 [32]	United States	65	7.5 or 12	An author of the study	Surgical and pathological findings
Lee 2005 [33]	United Kingdom	301	7.5 or 12	Experienced radiologists	Surgery, histopathology or cytology

NCD: not clearly described

**Table 3:** Characteristics of the twenty-two included CT studies

Study **(year**, reference)	County	No. of patients	Use of intravenous contrast (dose)	Section thickness (mm), gap (mm)	Interpreter(s)	Reference standard
Kim 1997 [21]	South Korea	95	NCD	10, 10	Two radiologists independently	Surgical and pathological findings
Stell 1996 [22]	United Kingdom	103	NCD	NCD	Experienced personnel	Histological examination
Asencio 1997 [23]	Germany	71	NCD	NCD	NCD	Surgical and histologic evaluation
Ozmen 2003 [27]	United States	48	NCD	NCD	NCD	Histological examination
Nozoe 1999 [28]	United States	36	NCD	NCD	An experienced gynecologist	Operation findings
Kayaalp 2002 [29]	United Kingdom	118	NCD	10, 10	A consultant radiologist	Histopathological examinations
Lim 2006 [34]	South Korea	124	60% iodinated contrast material (2 mL/kg)	1.0-1.5 or 3.0-7.0, NCD	Three experienced gastrointestinal radiologists	Surgical and histopathologic standards
Chen 2005 [36]	United States	68	60% iodine (maximum 150 mL)	7, NCD	An abdominal radiologist	Surgical and histological classification
Chamadol 2008 [39]	Thailand	64	Iodinated contrast material (100 mL)	8, NCD	An experienced radiologist	Surgical-pathologic results
Yajima 2006 [40]	United States	413	Iodinated contrast material (NCD)	10, NCD	Expert radiologists	Clinical, surgical reports, histopathologic findings
Yun 2005 [41]	United States	81	NCD (2 mL/kg)	3-5, NCD	NCD	Histopathologic examination
Kim 2005 [43]	United States	124	Iopromide (150 ml)	5.0, NCD	Two experienced gastrointestinal radiologists	Histopathologic analysis
D'Elia 2000 [44]	Germany	127	Non-ionic contrast medium (200 ml)	10,10	Two radiologists	Histopathologic staging
Adachi 1997 [45]	United States	56	Loparimon or omnipaque (100 ml)	NCD, NCD	One radiologist	Surgical and histological diagnosis
Shinohara 2005 [46]	Japan	112	Non-ionic contrast medium (100 ml)	2.5, 2.5	Two authors of the study	Surgical and histological diagnosis
Davies 1997 [47]	United Kingdom	105	Ultravist (150 ml)	10, 5	One radiologist	TNM histopathological stage
Yan 2007 [48]	China	220	Non-ionic contrast medium (1.5 ml/kg)	3.75-5, NCD	Two radiologists	Surgical and histological diagnosis
Roic 1994 [49]	Slovenia	45	Ioxitalamate (100 ml)	8, NCD	NCD	Surgical and pathological finding
Gamón 2002 [50]	Spain	50	non-ionic iodated contrast medium (120 ml)	5, 4	A single experienced radiologist	Surgical and pathological diagnosis
Zhang 2002 [51]	China	43	Cardiografin (80-100 ml)	5-10, 5-10	Two radiologists	Surgical and pathological examination
Yan 2010 [52]	China	640	Iopromide (180 ml)	5, 2.5	Two radiologists	Surgical and pathological findings
Pan 2010 [53]	China	350	Iopromide (180 ml)	5, NR	Two experienced physicians	Surgical and pathological findings

NCD: not clearly described.

**Table 4:** Characteristics of the two included MRI studies

Study	County	No. of patients	Use of intravenous contrast (dose)	Section thickness **(mm)**, gap (mm)	Field strength (T), coil type	Interpreter(s)	Reference Standard
Tang 2006 [37]	China	25	Gadolinium, 0.1 mmol/kg	10, NCD	0.5, array body coil	Two experienced MRI specialists	Surgical and histopathologic examination
Li 2007 [38]	China	35	Gadolinium, 0.1 mmol/kg	NCD, NCD	1.5, Phased array body coil	Two experienced radiologists	Surgical and histopathologic examination

NCD: not clearly described

**Table 5:** Characteristics of the five included 18F-FDG PET studies

Study (year, reference)	County	No. of patients	Time of fasting before scanning	FDG dose, time interval between FDG administration and scanning	Attenuation **correction**, reconstruction method	Interpreter(s)	Reference standard
Lim 2006 [34]	South Korea	124	4h	370-555 MBq, 60 min	Yes, order subset expectation maximization	Two experienced nuclear medicine physicians	Surgical and histopathologic standards
Yoshioka 2003 [35]	United States	20	4 h	Mean 222 MBq, 30 min	Yes, NCD	Three PET specialists	CT, cytology, and clinical course
Yoshioka 2003 [35]	United States	22	4 h	Mean 222 MBq, 45 min	Yes, NCD	Three PET specialists	CT, cytology, and clinical course
Chen 2005 [36]	United States	68	4 h	370-555 Mbq, 60 min	Yes, iterative	Two experienced nuclear medicine physicians	Surgical and histological classification
Yun 2005 [41]	United States	81	4 h	370 MBq, 60 min	Yes, iterative	Two experienced nuclear medicine physicians	Histopathologic examination
Yeung 1998 [42]	United States	23	6 h	370 MBq, 45 min to 1 h	Yes, NCD	An experienced PET reader	Histology, surgical findings, clinical follow-up

NCD: not clearly described.

The quality of included studies was assessed based on the 14 items of QUADAS (Table 6). The total score varied from 14 to 22 in US studies, 17 to 25 in EUS studies, 14 to 23 in CT studies, 15 to 19 in MRI studies, and 16 to 21 in ^18^F-FDG PET studies.

**Table 6:** Quality assessment of included studies

Imaging modality	Study (year, reference)	Criteria of quality assessment														
		
		1	2	3	4	5	6	7	8	9	10	11	12	13	14	TS
US and CT	Kim 1997 [21]	+	+	+	+	+	+	+	+/-	+/-	+/-	+/-	+/-	+/-	+	22
US and CT	Stell 1996 [22]	+	+	+	+/-	+	+	+	+/-	+/-	+	+/-	+/-	+/-	+	22
US and CT	Asencio 1997 [23]	+	+	+	+/-	+	+	+	-	+/-	+/-	+/-	+/-	+/-	-	18
US	Possik 1986 [24]	+	+	+	+/-	-	+	+	-	+/-	+/-	+/-	+/-	+/-	-	16
US	Derchi 1983 [25]	+	+	+	+/-	-	-	+	+	+/-	+	+/-	+/-	+/-	+	19
US	Liao 2004 [26]	+	-	+	+/-	-	-	+	+	+/-	+/-	+/-	+/-	+/-	-	14
US, EUS and CT	Ozmen 2003 [27]	+	+	+	+/-	-	-	+	-	-	+	+	+/-	+/-	+	17
CT	Nozoe 1999 [28]	-	+	+	+/-	-	-	+	+/-	+/-	+/-	+/-	+/-	+/-	+	15
US and CT	Kayaalp 2002 [29]	+	-	+	+/-	+/-	-	+	+/-	+/-	+/-	+/-	+/-	+/-	+	16
EUS	Chu 2004 [30]	+	+	+	+	+	+	+	+	+/-	+	+	+/-	+/-	+	25
EUS	Tio 1990 [31]	-	+/-	+	+	+	-	+	+	+/-	+	+/-	+/-	+/-	+	19
EUS	Chen 2002 [32]	+/-	+/-	+	+	-	-	+	+	+/-	+/-	+/-	+/-	+/-	+	17
EUS	Lee 2005 [33]	+	-	+/-	-	-	+	+/-	+	+/-	+	+	+/-	+/-	+	17
CT and PET	Lim 2006 [34]	+	-	+	+/-	-	-	+	+	+	+/-	+/-	+/-	+/-	+	17
PET	Yoshioka 2003 [35]	+/-	-	+/-	+/-	+/-	+/-	+	+	+/-	+	+/-	+/-	+/-	+	17
CT and PET	Chen 2005 [36]	+	+/-	+	+/-	+/-	+/-	+	+	+	+	+/-	+/-	+/-	+	21
MRI	Tang 2006 [37]	-	+/-	+	+	+/-	+/-	+	+	+/-	+	+/-	+/-	+/-	+	19
MRI	Li 2007 [38]	-	+/-	+	+	+/-	+/-	+	+	+/-	+	+/-	+/-	+/-	-	15
CT	Chamadol 2008 [39]	+/-	+/-	+	-	-	-	+	+	+/-	+	+/-	+	+/-	+	17
CT	Yajima 2006 [40]	-	+/-	+	+/-	-	+/-	+	+	+/-	+	+/-	+/-	+/-	+	17
CT and PET	Yun 2005 [41]	+	+/-	+	+/-	-	-	+/-	+/-	+/-	+	+	+/-	+/-	+	17
PET	Yeung 1998 [42]	+/-	+/-	+	+/-	-	-	+	+	+/-	+/-	+/-	+/-	+/-	+	16
CT	Kim 2005 [43]	+/-	+/-	+	+	-	-	+	+	+/-	+	+/-	+/-	+/-	+	18
CT	D'Elia 2000 [44]	+/-	+/-	+	+/-	-	-	+	+	+/-	+/-	+/-	+/-	+/-	+	16
CT	Adachi 1997 [45]	-	+/-	+	+/-	+	+/-	+	+/-	+/-	+/-	+/-	+/-	+/-	+	17
CT	Shinohara 2005 [46]	-	+/-	+	+	+	+	+	+	+/-	+	+/-	+/-	+/-	+	21
CT	Davies 1997 [47]	+/-	+/-	+	+	+	+	+	+	+	+	+/-	+/-	+/-	+	23
CT	Yan 2007 [48]	+	-	+	+	-	-	+	+/-	+/-	+	+/-	+/-	+/-	+	17
CT	Roic 1994 [49]	+/-	-	+	+/-	-	+	+	+/-	+/-	+/-	+/-	+/-	+/-	-	14
CT	Gamón 2002 [50]	+/-	-	+	+	+/-	+/-	+	+	+/-	+/-	+/-	+/-	+/-	+	18
CT	Zhang 2002 [51]	+	-	+	+/-	+/-	+/-	+	+	+/-	+/-	+/-	+/-	+/-	+	18
CT	Yan 2010 [52]	+	+/-	+	+	+	+	+	+	+/-	+	+/-	+/-	+/-	+	23
CT	Pan 2010 [53]	+	+/-	+	+/-	+	+	+	+/-	+/-	+/-	+/-	+/-	+/-	+	20

TS: total score.

### Results of meta-analysis

#### Heterogeneity tests

Table 7 presented the Spearman correlation coefficient for each test. The *p *value was larger than 0.1 except for EUS in the detection of peritoneal metastasis.

**Table 7:** Spearman correlation coefficient Logit (sensitivity) vs Logit (1- specificity)

Outcomes	Liver metastasis			Peritoneal metastasis			
	
	US	CT	PET	US	EUS	CT	PET
Scc	0.643	-0.143	0.400	0.200	1.000	0.329	0.200
p-value	0.119	0.598	0.600	0.800	0.000	0.297	0.800

Scc: Spearman correlation coefficient

Table 8 presented the results of Cochrane-Q test. For EUS in the detection of peritoneal metastasis, Cochrane-Q test failed to be conducted using the metandi command in stata software due to unstability. Except for US and PET in the detection of peritoneal metastasis, the *p *value of Cochrane-Q test was less than 0.1, which suggested significant heterogeneity between included studies.

**Table 8:** Results of Cochrane-Q test

Outcomes		Liver metastasis			Peritoneal metastasis			
		US	CT	PET	US	EUS	CT	PET
Sen	Q-value	23.87	40.96	7.95	7.24	FC	82.07	6.12
	p-value	0.00	0.00	0.09	0.12	FC	0.00	0.19
Spe	Q-value	153.95	77.99	19.61	5.34	FC	57.09	34.25
	p-value	0.00	0.00	0.00	0.25	FC	0.00	0.00
DOR	Q-value	88.72	40.12	30.55	18.90	FC	23.50	44.18
	p-value	0.00	0.00	0.00	0.00	FC	0.05	0.00

Sen: sensitivity; Spe: Specificity; DOR: diagnostic odds ratio; FC: failed to calculate

The results were calculated by using the midas command in stata software.

### Diagnostic value

#### Liver metastasis

The data were available in 8 US studies [21-27,29], 2 EUS studies [27,31], 18 CT studies [21-23,27,29,36,39,41,43-51,53], 2 MRI studies [37,38], and 4 ^18^F-FDG PET studies [35,36,41,42]. Meta-analysis was based on the bivariate model in the presence of significant heterogeneity.

Pooled sensitivity for US, CT and ^18^F-FDG PET in detecting liver metastasis were 0.54 (95% CI: 0.34-0.73), 0.74 (95% CI: 0.59-0.85) and 0.70 (95% CI: 0.36-0.90) respectively (Table 9).

**Table 9:** Results of diagnostic value of imaging

Imaging modality		Sen (95%CI)	Spe (95%CI)	DOR (95%CI)
Liver metastasis	US	0.54 (0.34-0.73)	0.98 (0.90-0.99)	50.25 (13.48-187.32)
	CT	0.74 (0.59-0.85)	0.99 (0.97-1.00)	251.14 (83.53-755.07)
	PET	0.70 (0.36-0.90)	0.96 (0.81-0.99)	56.46 (8.47-376.23)
Peritoneal metastasis	US	0.09 (0.03-0.21)	0.99 (0.96-1.00)	10.63 (1.54-73.36)
	EUS	0.34 (0.10-0.69)	0.96 (0.87-0.99)	13.07 (6.42-26.62)
	CT	0.33 (0.16-0.56)	0.99 (0.98-1.00)	66.18 (27.28-160.53)
	PET	0.28 (0.17-0.44)	0.97 (0.83-1.00)	12.49 (2.22-70.10)

Sen: sensitivity; Spe: Specificity; DOR: diagnostic odds ratio; CI: confidence interval.

The results were combined using the metandi command (based on bivariate model) in stata software.

Pooled specificity for US, CT and ^18^F-FDG PET in detecting liver metastasis were 0.98 (95% CI: 0.90-0.99), 0.99 (95% CI: 0.97-1.00) and 0.96 (95% CI: 0.81-0.99), respectively. (Table 9)

Pooled DOR for US, CT and 18F-FDG PET in detecting liver metastasis were 50.25 (95% CI: 13.48-187.32), 251.14 (95% CI: 83.53-755.07) and 56.46 (95% CI: 8.47-376.23) respectively (Table 9).

Only two studies' data were sufficient for EUS and MRI, and we did not conduct pooled analysis, but presented the result of each study in Table 10.

**Table 10:** Results of EUS and MRI in the detection of liver metastases

Imaging modality	Study ID	Sen (95% CI)	Spe (95% CI)	DOR (95% CI)
EUS	Ozmen 2003 [27]	0.00 (0.00-0.46)	0.86 (0.71-0.95)	0.43 (0.02-8.63)
	Tio 1990 [31]	0.67 (0.09-0.99)	0.95 (0.88-0.99)	38.50 (2.85-519.60)
MRI	Tang 2006 [37]	1.00 (0.40-1.00)	1.00 (0.89-1.00)	567.00 (9.95-32300.14)
	Li 2007 [38]	1.00 (0.40-1.00)	1.00 (0.87-1.00)	477.00 (8.35-27250.55)

Sen: sensitivity; Spe: Specificity; DOR: diagnostic odds ratio; CI: confidence interval.

#### Peritoneal metastasis

The data were available in 5 US studies [21-23,25,29], 4 EUS studies [30-33], 15 CT studies [21,22,29,34,36,39,40,43-48,52,53] and 4 ^18^F-FDG PET studies [34-36,42]. Meta-analysis was based on the bivariate model in the presence of significant heterogeneity.

Pooled sensitivity for US, EUS, CT and ^18^F-FDG PET in detecting peritoneal metastasis were 0.09 (95% CI: 0.03-0.21), 0.34 (95% CI: 0.10-0.69), 0.33 (95% CI: 0.16-0.56) and 0.28 (95% CI: 0.17-0.44) respectively (Table 9).

Pooled specificity for US, EUS, CT and ^18^F-FDG PET in detecting peritoneal metastasis were 0.99 (95% CI: 0.96-1.00), 0.96 (95% CI: 0.87-0.99), 0.99 (95% CI: 0.98-1.00) and 0.97 (95% CI: 0.83-1.00), respectively (Table 9).

Pooled DOR for US, EUS, CT and ^18^F-FDG PET in detecting peritoneal metastasis were 10.63 (95% CI: 1.54-73.36), 13.07 (95% CI: 6.42-26.62), 66.18 (95% CI: 27.28-160.53) and 12.49 (95% CI: 2.22-70.10), respectively (Table 9).

#### HSROC curves

**Figure 2:**
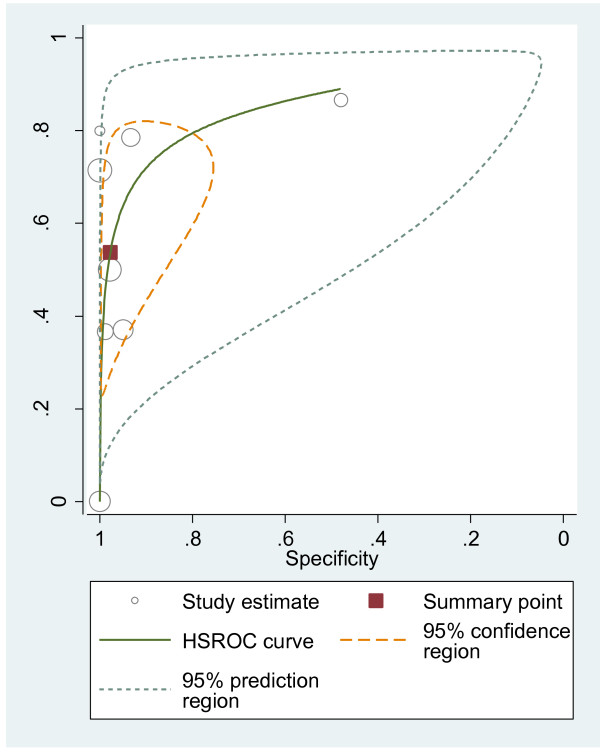
HSROC curve of US for the detection of liver metastases.

**Figure 3:**
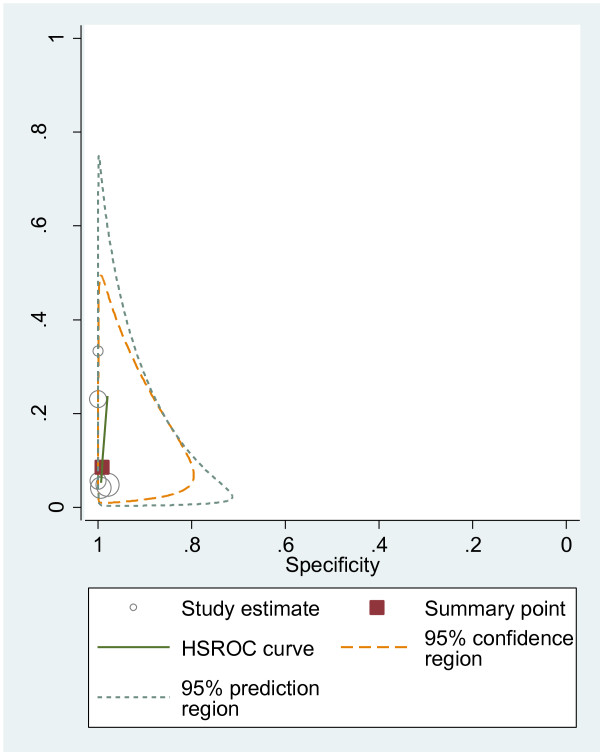
HSROC curve of US for the detection of peritoneal metastases.

**Figure 4:**
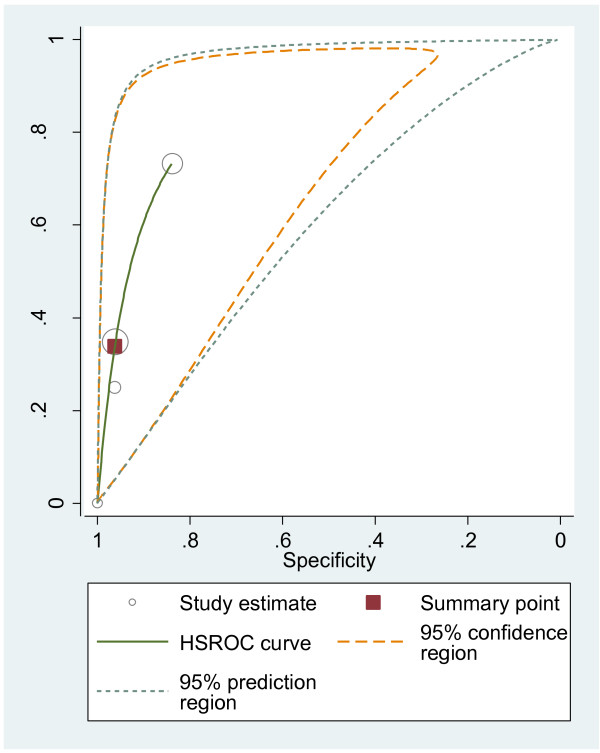
HSROC curve of EUS for the detection of peritoneal metastases.

**Figure 5:**
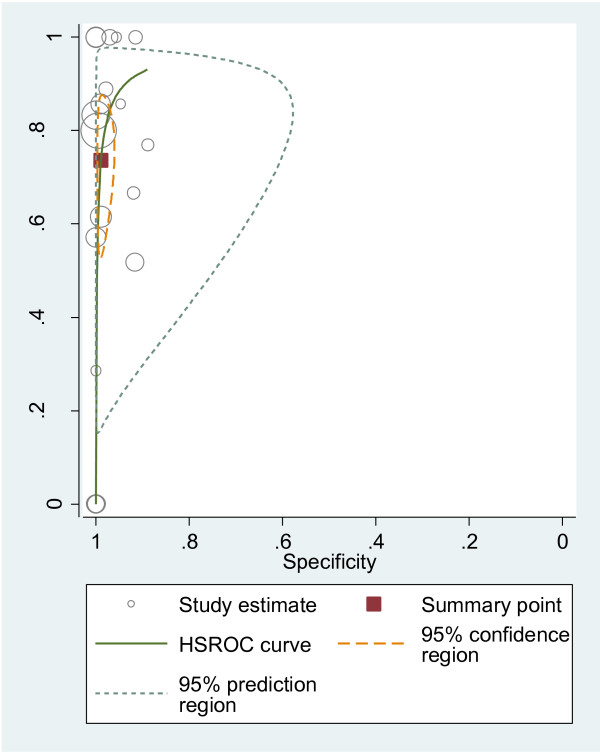
HSROC curve of CT for the detection of liver metastases.

**Figure 6:**
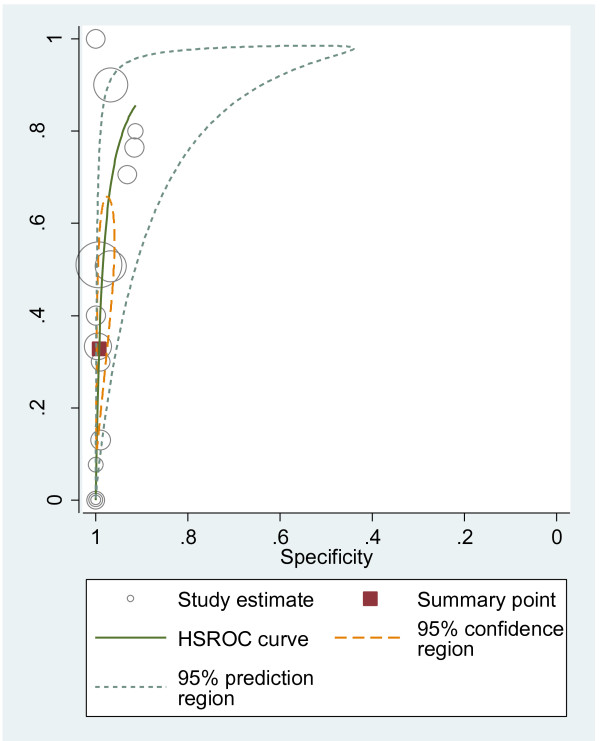
HSROC curve of CT for the detection of peritoneal metastases.

**Figure 7:**
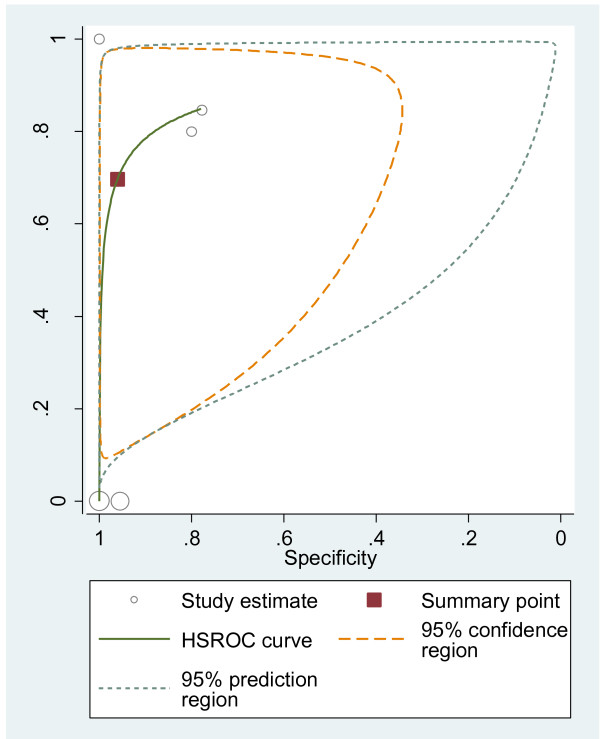
HSROC curve of 18F-FDG PET for the detection of liver metastases.

**Figure 8:**
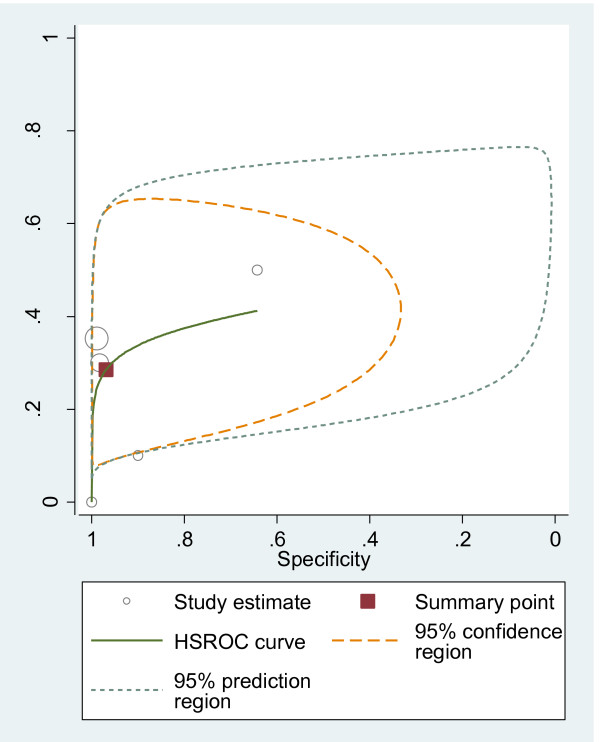
HSROC curve of 18F-FDG PET for the detection of peritoneal metastases.

We plotted HSROC curves to graphically present the results (Figure 2, 3, 4, 5, 6, 7, 8). In HSROC curves, the index test's sensitivity (true positive rate) was plotted on the y axis against 1-specificity (false negative rate) on the x axis. In addition, the 95% confidence region and a 95% prediction region around the pooled estimates were plotted to illustrate the precision with which the pooled values were estimated (confidence ellipse of a mean) and to show the amount of between study variation (prediction ellipse; the likely range of values for a new study) [16].

#### Publication bias

**Figure 9:**
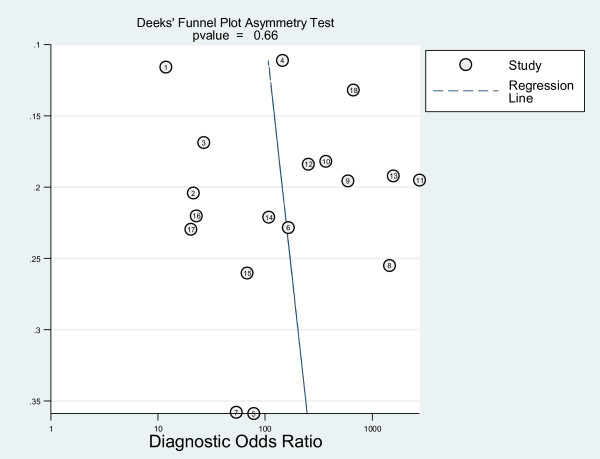
Funnel plot based on the data of CT for the detection of liver metastases.

Because the number of included studies was few, we only explored publication bias using the data of CT in detecting liver metastasis, which included 18 studies. As a result, the funnel plot seemed symmetrical with a *P *value of 0.66, and this suggested a low risk of publication bias (Figure 9).

## Discussion

As far as we know, this systematic review is the first study that evaluates the summary estimates of sensitivity and specificity of five imaging modalities which are currently used for the detection of hepatic and peritoneal metastases. The pooled result basing on the bivariate model showed that CT is the most sensitive imaging method [0.74 (95% CI: 0.59-0.85)] with a high rate of specificity [0.99 (95% CI: 0.97-1.00)] in detecting hepatic metastasis, and EUS is the most sensitive imaging modality [0.34 (95% CI: 0.10-0.69)] with a specificity of 0.96 (95% CI: 0.87-0.99) in detecting peritoneal metastasis.

The strengths of this systematic review were its well-defined search strategy, selection of study according to the strict inclusion criteria, independent methodological quality assessment by two reviewers and more valid statistical model for diagnostic meta-analysis in the presence of heterogeneity. Of course, our study was not faultless. Firstly, some included studies had a low methodological quality. For instance, the two eligible MRI studies only included patients with advanced gastric cancer, this might lead to bias by non-representative patient spectrum. Additionally, the great mass of included studies had a potential bias of partial verification, which was interpreted as not all of the study group receive confirmation of the diagnosis by the reference standard. It was especially noted that all included studies did not report the uninterpretable/intermediate test results, which might lead to the biased assessment of the test characteristics. Secondly, except for CT studies, the number of eligible studies is less. Besides many clinical characteristics of patients (such as Lauren classification) and technology parameters of imaging (such as slice Thickness and interslice gap of CT) were mixed or missing in included studies, so we failed to perform subgroup analysis or meta-regression, which might find out other possible causes of heterogeneity. Thirdly, due to few eligible studies, we only used the data of CT in detecting liver metastasis to test publication bias, therefore potential bias might occur. Fourthly, heterogeneity caused by threshold/cut off effect was present for EUS in detecting peritoneal metastasis, thus we should interpreted the pooled results prudently.

DOR, which means the ratio of the odds of positive test results in diseased group relative to the odds of positive test results in non-diseased group, is considered as another important indicator of test accuracy that combines the data from sensitivity and specificity into a new index [54]. The value of a DOR ranges from zero to infinity, with higher value indicating higher accuracy. A DOR of 1.0 shows that a test can not distinguish between patients with the disease and those without it. We found that CT seemed to be more helpful in the detection of liver and peritoneal metastases with a pooled DOR 251.14 (95% CI: 83.53-755.07) and 66.18 (95% CI: 27.28-160.53) respectively. Unlike traditional SROC plots, HSROC curves was plotted based on hierarchical models in our meta-analysis, which clearly presented the result of a global summary of test performance, the 95% confidence ellipse around the mean values of sensitivity and specificity of radiographer reporting, as well as a 95% prediction ellipse for individual value of sensitivity and specificity.

Previous studies reported that sensitivity of US ranged from 0.36-0.87 in detecting liver metastasis [22,25,27] and 0.05-0.33 in detecting peritoneal metastasis [25,29]. Then most of these studies excluded patients with obvious distant metastases, which might be the reason of low sensitivity of US in detecting liver and peritoneal metastases. We found that the pooled sensitivity of US in assessing hepatic metastasis was as low as 0.54 (95% CI: 0.34-0.73) with an acceptable specificity of 0.98 (95% CI: 0.90-0.99), and the pooled sensitivity in detecting peritoneal metastasis was 0.09 (95% CI: 0.03-0.21) with a relatively high specificity of 0.99 (95% CI: 0.96-1.00). This showed that US was more helpful in patients suspected of liver or peritoneal metastases.

EUS was initially developed primarily to overcome the limitations of abdominal ultrasonography in pancreas and a number of studies provided evidences on the high diagnostic accuracy and important role of EUS in staging of gastric cancer [55]. Compared with abdominal US, EUS has the advantage of placing the transducer close to the lesion without interference of bowel gas, bone or fat. However, EUS has an inherent disadvantage of operator dependency, which was the same as abdominal US. Although it is well suitable for the assessment of T staging, EUS has a limited effect in the overall assessment of more distant spread. Additionally, EUS is an invasive technique requiring sedation which will cause possible sedation-related complications [11]. Previous studies mainly focused on the usefulness of EUS in the evaluation of local invasion and LN staging [11,12]; but studies aiming at assessing the liver and peritoneal metastases were few [30-33]. We did not perform combined analysis using the data of EUS in the evaluation of liver metastases in the case of only two eligible studies. Although the result of our meta-analysis indicated that EUS was the most sensitive imaging modality in detecting peritoneal metastasis, the combined sensitivity was as low as 0.34 (95% CI: 0.10-0.69). This was similar with another systematic review, which concluded that EUS is not designed to look at distant metastasis [56]. It was reported that laparoscopy facilitated detection of EUS or CT-occult micrometastases on the peritoneal surface or in the liver [57], and identified EUS or CT-occult metastatic disease in 23% to 37% of patients [24,58]. The result suggested that laparoscopy should be integrated as part of the recommended staging algorithm in the detection of liver and peritoneal metastases in aftertime.

Abdominal CT can demonstrate not only the stomach wall and the adjacent tissue, but also the presence of distant metastases by providing rapid and high spatial resolution imaging [21,59]. The sensitivity of CT in the detection of liver metastasis ranged from 0.29-1.00 in included studies [21-24,27-29,44-51,53], the cause of so wide an interval possibly was the use of different tomography techniques in included studies, for example some researchers used single-detector row CT but others used multi-detector CT (MDCT), which can overcome the low scanning speed of single-detector row CT by its ability to make thinner sections in a shorter time. In our study we found that the diagnostic accuracy of CT was moderate with a pooled sensitivity of 0.74 (95% CI: 0.59-0.85) in the detection of liver metastasis. Additionally, the result of meta-analysis indicated that the sensitivity of CT in detecting peritoneal metastasis was very low [0.33 (95% CI: 0.16-0.56)], which supported the viewpoint that peritoneal metastasis was one of the limitations of CT in predicting the stage of gastric cancer preoperatively [21]. Newer MDCT technology (such as the application of 128- to 256-section MDCT scanners or dual-source technology) was reckoned to improve diagnostic performance with a spatial resolution of 5 mm or less in diameter [12]. Compared with CT, PET has an advantage of providing functional information. Currently PET is not only being evaluated as a staging tool for gastric cancer, but also useful for monitoring tumor recurrence and response to neoadjuvant therapy [59,60]. Although some researchers reported that PET had utilities in detecting liver and peritoneal metastases (the sensitivity could achieve 100% and 57% respectively) [42,61], we did not found that ^18^F-FDG PET had advantages over CT in the assessment of liver and peritoneal metastases in our meta-analysis. Possible reasons for the reported low to moderate sensitivity of FDG-PET is lack of detailed anatomic information in the area of significant tracer uptake and its limited resolution. It was reported that combining both PET and CT (PET/CT) has demonstrated further improvements in diagnostic accuracy recently [59]. The method unites the high anatomic spatial information from CT with the functional information offered by PET, and has a benefit of the rapid CT based attenuation correction of PET. This can decrease scanning time and increase the degree of comfort. Pyrimidine analog 3-deoxy-3-18F-fluorothymidine (^18^F-FLT), a new stable PET tracer was used for improving the diagnostic accuracy lately. It was reported that ^18^F-FLT had a higher sensitivity than ^18^F-FDG PET in the detection of locally advanced gastric cancer [62]. However, whether this imaging modality will improve the diagnostic accuracy of liver and peritoneal metastases needs further investigation.

MRI has evolved to be an important imaging method for detection and characterization of most of common diseases of the abdomen including gastric cancer [63,64]. Advantages of MRI over CT include the ability of generating significantly greater soft tissue contrast resolution, and the ability of removing the risk of iodinated contrast-induced nephropathy or ionizing radiation [64]. However only two eligible MRI studies [37,38] were identified in our review, and all from China, therefore the data were not combined. The two studies found that MRI had both high sensitivity and specificity in detecting liver metastasis. Some researchers reported that diffusion-weighted (DW) MRI was more sensitive than CT in detecting liver and peritoneal metastases, and functional parameters such as apparent diffusion coefficient (ADC) could monitor the response to neoadjuvant chemotherapy [65]. These results seemed inspiring, whereas the sample size was small and methodological quality was moderate. Therefore more MRI (especially DW-MRI) studies focusing on evaluating the liver and peritoneal metastases are urgently needed in future.

## Conclusions

Although the result of our meta-analysis showed that CT was the most sensitive imaging method with a high rate of specificity in detecting hepatic metastasis, and EUS was the most sensitive imaging modality with a relatively low rate of specificity (compared with the three other imaging methods) in detecting peritoneal metastasis, we concluded that US, EUS, CT and ^18^F-FDG PET did not obtain consistently high sensitivity and specificity in detecting liver and peritoneal metastases in patients with gastric cancer. More attention should be paid to laparoscopy, PET/CT, DW-MRI, as well as new PET tracers such as ^18^F-FLT in the detection of liver and peritoneal metastases of gastric cancer in future.

## Competing interests

The authors declare that they have no competing interests.

## Authors' contributions

ZW and JQC contributed equally to this article. ZW and JQC designed this study; ZW and Chen JQ performed this research; ZW and JQC analyzed the data; JQC interpreted the results; ZW drafted the manuscript; JQC revised the paper. The two authors both approved the final manuscript.

## Appendix

**Midas**: is a comprehensive program of statistical and graphical routines for undertaking meta-analysis of diagnostic test performance in Stata. It facilitates exploratory analysis of heterogeneity, publication and other precision-related biases.

**Metandi**: The metandi command display the results in two alternative parameterizations and produce a customizable plot. It also displays some familiar summary measures (such as sensitivity and specificity). The command requires either Stata 10 or above (which has the new command xtmelogit), or Stata 8.2 or above with gllamm installed.

1. Midas command for testing the heterogeneity of sensitivity and specificity:

midas tp fp fn tn, texts(0.60) bfor(dss) ford fors

2. Midas command for testing the heterogeneity of DOR:

midas tp fp fn tn, texts(0.60) bfor(dlor) ford fors

3. Metandi command for the pooled analysis of sensitivity, specificity and DOR, and for plotting the HSROC curves:

metandi tp fp fn tn, plot

4. Midas command for testing publication bias:

midas tp fp fn tn, pubbias

(tp: true positives, fp: false positives, fn: false negatives, tn: true negatives)

## Pre-publication history

The pre-publication history for this paper can be accessed here:

http://www.biomedcentral.com/1471-230X/11/19/prepub

## References

[B1] Parkin DM Bray F Ferlay J Pisani P Global cancer statistics, 2002 CA Cancer J Clin 2005 55 74 108 10.3322/canjclin.55.2.74 15761078

[B2] Crew KD Neugut AI Epidemiology of gastric cancer World J Gastroenterol 2006 12 354 362 16489633 10.3748/wjg.v12.i3.354 PMC4066052

[B3] Roukos DH Kappas AM Perspectives in the treatment of gastric cancer Nat Clin Pract Oncol 2005 2 98 107 10.1038/ncponc0099 16264882

[B4] Sakamoto Y Ohyama S Yamamoto J Yamada K Seki M Ohta KI Kokudo N Yamaguchi T Muto T Makuuchi M Surgical resection of liver metastases of gastric cancer: An analysis of a 17-year experience with 22 patients Surgery 2003 33 507 511 10.1067/msy.2003.147 12773978

[B5] Koga S Kawaguchi H Kishimoto H Therapeutic significance of noncurative gastrectomy for gastric cancer with liver metastasis Am J Surg 1980 140 356 359 10.1016/0002-9610(80)90167-1 6158880

[B6] Okano K Maeba T Ishimura K Karasawa Y Goda F Wakabayashi H Usuki H Maeta H Hepatic resection for metastatic tumors from gastric cancer Ann Surg 2002 235 86 91 10.1097/00000658-200201000-00011 11753046 PMC1422399

[B7] Yamada E Miyaishi S Nakazato H The surgical treatment of cancer of the stomach Int Surg 1980 65 387 399 6161095

[B8] Sadeghi B Arvieux C Glehen O Beaujard AC Rivoire M Baulieux J Fontaumard E Brachet A Caillot JL Faure JL Porcheron J Peix JL François Y Vignal J Gilly FN Peritoneal carcinomatosis from non-gynecologic malignancies: Results of the EVOCAPE 1 multicentric prospective study Cancer 2000 88 358 363 10.1002/(SICI)1097-0142(20000115)88:2<358::AID-CNCR16>3.0.CO;2-O 10640968

[B9] Moehler M Galle PR Gockel I Junginger T Schmidberger H The multidisciplinary management of gastrointestinal cancer. Multimodal treatment of gastric cancer Best Pract Res Clin Gastroenterol 2007 21 965 81 10.1016/j.bpg.2007.10.003 18070698

[B10] Power DG Schattner MA Gerdes H Brenner B Markowitz AJ Capanu M Coit DG Brennan M Kelsen DP Shah MA Endoscopic Ultrasound Can Improve the Selection for Laparoscopy in Patients with Localized Gastric Cancer J Am Coll Surg 2009 208 173 178 10.1016/j.jamcollsurg.2008.10.022 19228527

[B11] Kwee RM Kwee TC Imaging in local staging of gastric cancer: A systematic review J Clin Oncol 2007 25 2107 2116 10.1200/JCO.2006.09.5224 17513817

[B12] Kwee RM Kwee TC Imaging in assessing lymph node status in gastric cancer Gastric Cancer 2009 12 6 22 10.1007/s10120-008-0492-5 19390927

[B13] Polkowski M Endosonographic staging of upper intestinal malignancy Best Pract Res Clin Gastroenterol 2009 23 649 661 10.1016/j.bpg.2009.05.002 19744630

[B14] Whiting P Rutjes AWS Reitsma JB Bossuyt PMM Kleijnen J The development of QUADAS: A tool for the quality assessment of studies of diagnostic accuracy included in systematic reviews BMC Med Res Methodol 2003 3 1 13 10.1186/1471-2288-3-25 14606960 PMC305345

[B15] Arends LR Hamza TH Van Houwelingen JC Heijenbrok-Kal MH Hunink MGM Stijnen T Bivariate random effects meta-analysis of ROC curves Med Decis Making 2008 28 621 638 10.1177/0272989X08319957 18591542

[B16] Geersing GJ Janssen KJM Oudega R Bax L Hoes AW Reitsma JB Moons KG Excluding venous thromboembolism using point of care D-dimer tests in outpatients: A diagnostic meta-analysis BMJ 2009 339 b2990 10.1136/bmj.b2990 19684102 PMC2727580

[B17] Reitsma JB Glas AS Rutjes AWS Scholten RJPM Bossuyt PM Zwinderman AH Bivariate analysis of sensitivity and specificity produces informative summary measures in diagnostic reviews J Clin Epidemiol 2005 58 982 90 10.1016/j.jclinepi.2005.02.022 16168343

[B18] Deeks JJ Macaskill P Irwig L The performance of tests of publication bias and other sample size effects in systematic reviews of diagnostic test accuracy was assessed J Clin Epidemiol 2005 58 882 893 10.1016/j.jclinepi.2005.01.016 16085191

[B19] Dwamena BA Midas: computational and graphical routines for meta-analytical integration of diagnostic accuracy studies in Stata 2007 Division of NuclearMedicine, Department of Radiology, University of Michigan Medical School, Ann Arbor, Michigan

[B20] Harbord RM Metandi: Stata module for meta-analysis of diagnostic accuracy 2008 Statistical Software Components. Boston College, Departement of Economics

[B21] Kim JJ Jung HC Song IS Choi KW Kim CY Han JK Choi BI Park JG Lee KU Choe KJ Kim WH Preoperative evaluation of the curative resectability of gastric cancer by abdominal computed tomography and ultrasonography: a prospective comparison study Korean J Intern Med 1997 12 1 6 9159030 10.3904/kjim.1997.12.1.1 PMC4531968

[B22] Stell DA Carter CR Stewart I Anderson JR Prospective comparison of laparoscopy, ultrasonography and computed tomography in the staging of gastric cancer Br J Surg 1996 83 1260 1262 10.1002/bjs.1800830927 8983624

[B23] Asencio F Aguiló J Salvador JL Villar A De la Morena E Ahamad M Escrig J Puche J Viciano V Sanmiguel G Ruiz J Video-laparoscopic staging of gastric cancer. A prospective multicenter comparison with noninvasive techniques Surg Endosc 1997 11 1153 1158 10.1007/s004649900559 9373284

[B24] Possik RA Franco EL Pires DR Sensitivity, specificity, and predictive value of laparoscopy for the staging of gastric cancer and for detection of liver metastases Cancer 1986 58 1 6 10.1002/1097-0142(19860701)58:1<1::AID-CNCR2820580102>3.0.CO;2-K 2939942

[B25] Derchi LE Biggi E Rollandi GA Sonographic staging of gastric cancer AJR Am J Roentgenol 1983 140 273 276 6600341 10.2214/ajr.140.2.273

[B26] Liao SR Dai Y Huo L Yan K Zhang L Zhang H Gao W Chen MH Transabdominal ultrasonography in preoperative staging of gastric cancer World J Gastroenterol 2004 10 3399 3404 15526355 10.3748/wjg.v10.i23.3399 PMC4576217

[B27] Ozmen MM Zulfikaroglu B Ozalp N Ziraman I Hengirmen S Sahin B Staging laparoscopy for gastric cancer Surg Laparosc Endosc Percutan Tech 2003 13 241 244 10.1097/00129689-200308000-00003 12960785

[B28] Nozoe T Matsumata T Sugimachi K Usefulness of preoperative transvaginal ultrasonography for women with advanced gastric carcinoma Am J Gastroenterol 1999 94 2509 2512 10.1111/j.1572-0241.1999.01385.x 10484016

[B29] Kayaalp C Arda K Orug T Ozcay N Value of computed tomography in addition to ultrasound for preoperative staging of gastric cancer Eur J Surg Oncol 2002 28 540 543 10.1053/ejso.2002.1296 12217308

[B30] Chu KM Kwok KF Law S Wong KH A prospective evaluation of catheter probe EUS for the detection of ascites in patients with gastric carcinoma Gastrointest Endosc 2004 59 471 474 10.1016/S0016-5107(03)02873-6 15044880

[B31] Tio TL Coene PPLO Luiken GJHM Tytgat GNJ Endosonography in the clinical staging of esophagogastric carcinoma Gastrointest Endosc 1990 36 S2 S10 10.1016/S0016-5107(90)71008-5 2184081

[B32] Chen CH Yang CC Yeh YH Preoperative staging of gastric cancer by endoscopic ultrasound: The prognostic usefulness of ascites detected by endoscopic ultrasound J Clin Gastroenterol 2002 35 321 327 10.1097/00004836-200210000-00008 12352295

[B33] Lee YT Ng EKW Hung LCT Chung SCS Ching JYL Chan WY Chu WC Sung JJ Accuracy of endoscopic ultrasonography in diagnosing ascites and predicting peritoneal metastases in gastric cancer patients Gut 2005 54 1541 1545 10.1136/gut.2004.055772 15955787 PMC1774738

[B34] Lim JS Kim MJ Yun MJ Oh YT Kim JH Hwang HS Park MS Cha SW Lee JD Noh SH Yoo HS Kim KW Comparison of CT and 18F-FDG pet for detecting peritoneal metastasis on the preoperative evaluation for gastric carcinoma Korean J Radiol 2006 7 249 256 10.3348/kjr.2006.7.4.249 17143028 PMC2667611

[B35] Yoshioka T Yamaguchi K Kubota K Saginoya T Yamazaki T Ido T Yamaura G Takahashi H Fukuda H Kanamaru R Evaluation of 18F-FDG PET in patients with a, metastatic, or recurrent gastric cancer J Nucl Med 2003 44 690 699 12732669

[B36] Chen J Cheong JH Mi JY Kim J Joon SL Woo JH Sung HN Improvement in preoperative staging of gastric adenocarcinoma with positron emission tomography Cancer 2005 103 2383 2390 10.1002/cncr.21074 15856477

[B37] Tang QF Shen JK Feng YZ Wang GZ Zhang CY Qian MH Evaluation of dynamic 0.5T MRI in preoperative TNM-staging of advanced gastric carcinoma Chinese Journal of Medical Imaging Technology 2006 22 88 91

[B38] Li SF Zhao RF Li HB Li JD Jin JL Dynamic contrast enhanced MR study on preoperative TNM staging of advanced gastric carcinoma Chinese Journal of Medical Imaging Technology 2007 23 1187 1190

[B39] Chamadol N Wongwiwatchai J Bhudhisawasd V Pairojkul C Accuracy of spiral CT in preoperative staging of gastric carcinoma: Correlation with surgical and pathological findings J Med Assoc Thai 2008 91 356 363 18575289

[B40] Yajima K Kanda T Ohashi M Wakai T Nakagawa S Sasamoto R Hatakeyama K Clinical and diagnostic significance of preoperative computed tomography findings of ascites in patients with advanced gastric cancer Am J Surg 2006 192 185 190 10.1016/j.amjsurg.2006.05.007 16860627

[B41] Yun M Lim JS Noh SH Hyung WJ Cheong JH Bong JK Cho A Lee JD Lymph node staging of gastric cancer using18F-FDG PET: A comparison study with CT J Nucl Med 2005 46 1582 1588 16204706

[B42] Yeung HW Macapinlac H Karpeh M Finn RD Larson SM Accuracy of FDG-PET in Gastric Cancer. Preliminary Experience Clin Positron Imaging 1998 1 213 221 10.1016/S1095-0397(98)00018-1 14516555

[B43] Kim HJ Kim AY Oh ST Kim JS Kim KW Kim PN Lee MG Ha HK Gastric cancer staging at multi-detector row CT gastrography: Comparison of transverse and volumetric CT scanning Radiology 2005 236 879 885 10.1148/radiol.2363041101 16020558

[B44] D'Elia F Zingarelli A Palli D Grani M Hydro-dynamic CT preoperative staging of gastric cancer: Correlation with pathological findings. A prospective study of 107 cases Eur Radiol 2000 10 1877 1885 11305564 10.1007/s003300000537

[B45] Adachi Y Sakino I Matsumata T Iso Y Yoh R Kitano S Okudaira Y Preoperative assessment of advanced gastric carcinoma using computed tomography Am J Gastroenterol 1997 92 872 875 9149204

[B46] Shinohara T Ohyama S Yamaguchi T Muto T Kohno A Ogura T Kato Y Urashima M Preoperative TNM staging of advanced gastric cancer with multi-detector row computed tomography Japan Medical Association Journal 2005 48 175 182

[B47] Davies J Chalmers AG Sue-Ling HM May J Miller GV Martin IG Johnston D Spiral computed tomography and operative staging of gastric carcinoma: A comparison with histopathological staging Gut 1997 41 314 319 10.1136/gut.41.3.314 9378384 PMC1891482

[B48] Yan C Zhu ZG Yan M Chen KM Chen J Liu BY Yin HR Lin YZ Clinical significance of multi-slice spiral CT and serum tumor markers in the preoperative assessment of gastric carcinoma World Chinese Journal of Digestology 2007 15 3194 3203

[B49] Roic G Marotti M Zovak M Klaric R Krolo I Roic D Accuracy of preoperative CT scanning in staging of gastric carcinoma Radiother Oncol 1994 28 114 118

[B50] Gamón Giner R Escrig Sos J Salvador Sanchís JL Ruiz del Castillo J García Vila JH Marcote Valdivieso E Helical CT evaluation in the preoperative staging of gastric adenocarcinoma Rev Esp Enferm Dig 2002 94 597 600 12647409

[B51] Zhang HX Wang HY Shen BZ Qu LY Yu SJ The clinical value of CT in the diagnosis of gastric cancer Journal of Practical Oncology 2002 16 262 264

[B52] Yan C Zhu ZG Yan M Chen KM Chen J Xiang M Chen MM Liu BY Yin HR Lin YZ Value of multidetector-row CT in the preoperative prediction of peritoneal metastasis from gastric cancer: a single-center and large-scale study Chinese journal of gastrointestinal surgery 2010 13 106 110 20186619

[B53] Pan Z Zhang H Yan C Du L Ding B Song Q Ling H Huang B Chen K Determining gastric cancer resectability by dynamic MDCT European Radiology 2010 20 613 620 10.1007/s00330-009-1576-2 19707768

[B54] Glas AS Lijmer JG Prins MH Bonsel GJ Bossuyt PMM The diagnostic odds ratio: a single indicator of test performance J Clin Epidemiol 2003 56 1129 35 10.1016/S0895-4356(03)00177-X 14615004

[B55] Tamerisa R Irisawa A Bhutani MS Endoscopic ultrasound in the diagnosis, staging, and management of gastrointestinal and adjacent malignancies Med Clin North Am 2005 89 139 158 10.1016/j.mcna.2004.08.010 15527812

[B56] Puli SR Batapati Krishna Reddy J Bechtold ML Antillon MR Ibdah JA How good is endoscopic ultrasound for TNM staging of gastric cancers? A meta-analysis and systematic review World J Gastroenterol 2008 14 4011 4019 10.3748/wjg.14.4011 18609685 PMC2725340

[B57] Abdalla EK Pisters PWT Staging and preoperative evaluation of upper gastrointestinal malignancies Semin Oncol 2004 31 513 529 10.1053/j.seminoncol.2004.04.014 15297943

[B58] Feussner H Omote K Fink U Walker SJ Siewert JR Pretherapeutic laparoscopic staging in advanced gastric carcinoma Endoscopy 1999 31 342 347 10.1055/s-1999-28 10433041

[B59] Chin BB Clinical Utility of Combined 18F-Fluoro-2-deoxyglucose Positron Emission Tomography - Computed Tomography in the Evaluation of Gastrointestinal Malignancies Curr Med Imaging Rev 2008 4 255 269 10.2174/157340508786404107

[B60] Dassen AE Lips DJ Hoekstra CJ Pruijt JFM Bosscha K FDG-PET has no definite role in preoperative imaging in gastric cancer Eur J Surg Oncol 2009 35 449 455 19147324 10.1016/j.ejso.2008.11.010

[B61] Turlakow A Yeung HW Salmon AS Macapinlac HA Larson SM Peritoneal carcinomatosis: Role of 18F-FDG PET J Nucl Med 2003 44 1407 1412 12960184

[B62] Herrmann K Ott K Buck AK Lordick F Wilhelm D Souvatzoglou M Becker K Schuster T Wester HJ Siewert JR Schwaiger M Krause BJ Imaging gastric cancer with PET and the radiotracers 18F-FLT and 18F-FDG: A comparative analysis J Nucl Med 2007 48 1945 1950 10.2967/jnumed.107.044867 18006614

[B63] Chung JJ Semelka RC Martin DR Marcos HB Colon diseases: MR evaluation using combined T2-weighted single-shot echo train spin-echo and gadolinium-enhanced spoiled gradient-echo sequences Nucl Med Commun 2000 12 297 305 10.1002/1522-2586(200008)12:2<297::aid-jmri12>3.0.co;2-q 10931593

[B64] Martin DR Danrad R Herrmann K Semelka RC Hussain SM Magnetic resonance imaging of the gastrointestinal tract Top Magn Reson Imaging 2005 16 77 98 10.1097/01.rmr.0000179461.55234.7d 16314698

[B65] Roth Y Tichler T Kostenich G Ruiz-Cabello J Maier SE Cohen JS Orenstein A Mardor Y High-b-value diffusion-weighted MR imaging for pretreatment prediction and early monitoring of tumor response to therapy in mice Radiology 2004 232 685 692 10.1148/radiol.2322030778 15215551

